# Visualization and quantitative evaluation of aerosol deposition using 3D-printed adult nose cavities

**DOI:** 10.1016/j.heliyon.2024.e38179

**Published:** 2024-09-19

**Authors:** Wei He, Muhan Shi, Yaozhong Lu, Chengsheng Chu, Xiaolong Wang, Min Wang, Xiaofang Zhang

**Affiliations:** a Breath Medical Co., Ltd., Hefei, PR China; b Department of Otorhinolaryngology, Head and Neck Surgery, Peking University People's Hospital, Xi Zhi Men Nan Da Jie 11#, Beijing, 100044, PR China; c Lanzhou Institute of Chemical Physics, Chinese Academy of Sciences, No.18, Tianshui Middle Road, Lanzhou, PR China

**Keywords:** 3D nasal cavity, Visualization, Quantitative evaluation, Aerosol deposition, Drug delivery technique

## Abstract

Local steroid medication is one of the most important treatment options for chronic rhinosinusitis. Regional deposition has a higher clinical value compared with total deposition in predicting treatment outcomes or evaluating adverse reactions. The goal of this project is to propose an effective technique for visualizing and quantifying aerosol deposition in a three-dimensional adult nasal cavity, and to verify the practicality of this method. Three-dimensional (3D) nasal cavity models were constructed from computed tomography (CT) scans of one post-operative rhinosinusitis subject using imaging software. The nasal cast was coated with a water-indicating paste and deposited with saline; a liquid dressing was added to visualize the progress. The quantity of liquid dressing was evaluated via HPLC and the liquid deposition was analyzed within the nasal cast cavity. Herein, 98.77 % of the particles generated by the nebulizer were over 5 μm, suggesting that most of the aerosol could effectively enter the nasal cavity instead of the lower respiratory system. The liquid dressing was mainly deposited in the nasal cavity, ethmoid sinus, and frontal sinus according to the visualization tests. HPLC results suggested that the main deposits were the frontal sinus (up to 41.80 %) as well as in the sphenoid sinus and ethmoid sinus (14.00 %). The large particle nebulizer (BM-TCA) generally led to better deposition in sinus areas when compared to the smaller particle nebulizer (PARI). This technology allows for in vitro testing of various types of nasal preparations and equipment under various test methods.

## 1 Introduction

Chronic rhinosinusitis (CRS) is a common upper airway inflammatory disease affecting between 5.5 % and 28 % of the global population [1]. Routine treatment includes nasal irrigation, antibiotics, mucolytics, and corticosteroids. Systemic corticosteroids can cause many side effects and can only be used over the short term. Local steroids are an effective treatment and are a first-line management option for most CRS [2,3]. As the mucous membrane covered by a large surface area of nose's microvilli, the rich blood vessels and high permeability of the nasal mucosa facilitate systemic absorption. The venous blood in the nasal cavity is not affected by the first-pass effect of the liver [[4], [5], [6]]. However, the most challenging issue is how to deliver corticosteroids to the meatus nasi medius and frontal sinus. Aerosol drug delivery is one of the topical treatments for CRS and has attracted increasing attention. The progress of nasal administration therapy needs detailed and thorough development to guarantee the optimal delivery of drugs, including drug analysis [7], formulation optimization [3] and aerosol assesment [8].

Nasal models are used to evaluate the deposition of preparations in various sections of the cavities in nose areas. Owing to the shortage of verification and direct relevance to real-life medical situations, aerosol deposition patterns generated by computational models are limited in clinical practice [9]. Aerosol deposition in the nasal cavity has also been studied via animal models [[10], [11], [12]], cadaver specimens [13], and human subjects [[14], [15], [16], [17], [18]]. Wang's group used fluorescein in cadaver heads to analyze the effect of different head positions on drug deposition after frontal sinus surgery [13]. There are also studies using gamma scintillation with technetium-99m (^99m^Tc) radiolabeled particles to visualize the deposition distribution in the nose/nasal cast [[14], [15], [16], [17], [18], [19], [20], [21], [22]]. For these studies, traditional methods require a lot of mice and other experimental animals to establish animal models. These processes take a lot of time and money. The clinical research methods are also invasive which easily cause harm to the human body. To address this shortcoming, organ models were initially obtained by using conventional manufacturing techniques without the use of 3D-printed technique. However, the models produced by conventional manufacturing techniques cannot accurately exhibit the exact models and organizational states. With the continuous development of 3D-printed technique, high-precision biological tissue models can be manufactured more effectively at a lower cost than in the past. They allow the efficiency of nasal models to be tested in a more actual way than standard tests, while are cheaper and easier than clinical tests.

Recently, 3D-printed technique has made great progress [23]. In the field of biomedicine, 3D printing technology is a new research platform for biomaterials and artificial organs; it can produce complex 3D models [24,25]. Anatomically accurate nose model data reconstructed from CT (Computed Tomography) scans can fully replicate the nose model via 3D printing technology [26]. This technology offers high precision, simple equipment, and fast formation. It is very suitable for research and development of nasal nebulizers and drug formulations for nasal applications. The acquisition of research data depends on the nasal models used during the experiments. Some nasal models with simple structure can only be used for qualitative analysis and cannot be broken down into different components [27]. With the use of a digital camera and software, quantitative estimates of deposit areas are available in a monolithic nasal model. A correlation technique of Sar-Gel colorimetric and water mass was proposed to quantify the application of water from Sar-Gel images [27,28]. Besides the limitations of the test specimen, a precise methodology to quantitate and visualize aerosol deposition in the nasal cavity remains difficult. The following areas of interest are frequently studied: nostrils, vestibule, turbinates, olfactory region, and nasopharynx. Local or regional aerosol deposition/nasal drug delivery is closer to clinical results than total deposition in evaluating treatment results or adverse reactions [29]. There are many reports on the total deposition fraction (DF), while there are relatively few reports on effective methods for visualizing and quantifying regional or local deposition fractions [29]. Targeting aerosol deposition locally to very precise, spatially defined nasal regions is in its infancy. In vitro spray measurements listed by the US Food and Drug Administration (FDA) are controversial due to the failure of predicting human nasal deposition and bioequivalence [27,28]. Clearly, the FDA, pharmaceutical companies, and medical device companies lack a simple and reliable method to evaluate nasal aerosol deposition [27].

**Fig. 1:**
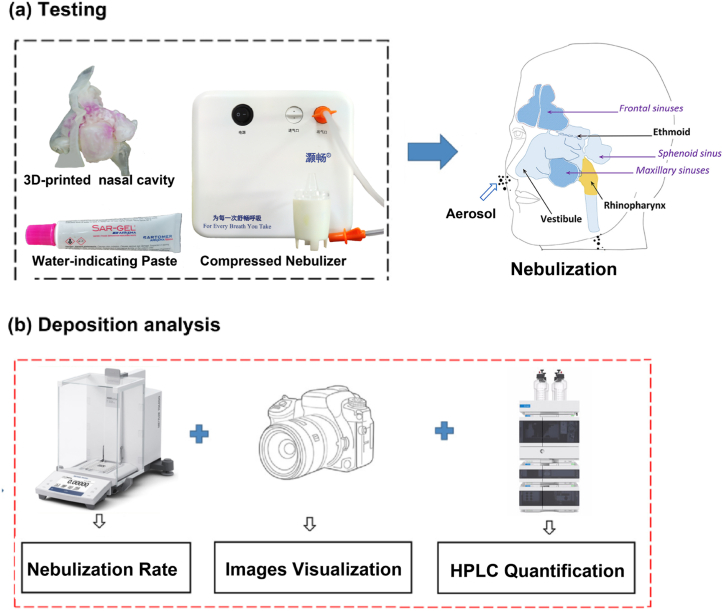
Intranasal deposition test. a) The 3D printed nose model, nebulizer and water-indicating paste were used for testing, and nebulizing process b) Deposition analyses were measured by nebulization rate, images visualization and HPLC quantification.

Therefore, the aim of this study was to design and conduct in vitro tests to visualize and quantify nasal aerosol deposition in high-precision 3D printing nose models (Fig. 1). A 3D-printed human nasal cavity was used and shown to completely replicate the anatomical structure of the nose (Fig. 1a). Delivered dosages (also referred to nebulization rate) were measured using a high-precision scale (Fig. 1b). A digital camera was used to visualize deposition areas by capturing the color changes (Fig. 1b). High-performance liquid chromatography (HPLC) methods were used to quantify the aerosol deposition (Fig. 1b). The aerosol deposition area could be easily and effectively visualized, quantified, and distinguished. This paper aimed to develop and verify an effective approach of visualizing and quantifying aerosol deposition in a 3D-printed adult nasal cavity.

## 2 Materials and methods

### 2.1 Materials

Sar-Gel® (Sartomer Company Inc.) a water-indicating paste was used to visualize the deposition and distribution of liquid dressings in the nasal cavity. Mometasone furoate (MF) reference substance (purity grade: 99.6 %) was acquired from the China National Institutes for Food and Drug Control (NIFDC). Mometasone furoate was obtained from KingYork (Tianjin, China). HPLC-grade methanol was obtained from Sigma (Shanghai, China). HPLC-grade methanol was obtained from Sigma (Shanghai, China). HPLC-grade acetonitrile was obtained from Sigma (Shanghai, China). The liquid dressing (mainly composed of PEG-400, propylene glycol) was developed and provided by Breath Medical Co., Ltd.

### 2.2 Nebulization rate of liquid dressing

A nebulizer with a liquid container (BM-TCA from Breath Medical Co., Ltd., free flow: 6 L/min nominal, pressure range: 200 kPa; PARI SINUS N from PARI GmbH, free flow: 4.6 L/min nominal, pressure range: 150 kPa) was used for nebulization. The rate of nebulization, referred to delivered dosages was calculated by Equation (1). Here, the weight of the liquid dressing before and after each nebulization was measured using an XS205DU analytical balance (Mettler Toledo, US) and recorded as m_1_ and m_2,_ respectively. The difference in weight was divided by the time (t, min) used for nebulization, indicating the dosages delivered by the nebulizer to the 3D model per minute.

Equation 1 $$\text{nebulization}\;\text{rate}=\left(m_{2}‐m_{1}\right)/t$$

### 2.3 Particle size distribution measurement

A HELOS-SPRAYER particle size analyzer (Sympatec GmbH) was used to analyze the particle size of the liquid dressing generated by the nebulizer (BM-TCA, or PARI). The instrument is equipped with a multi-element detector with a detection range of 0.25–875 μm. The liquid dressing or physiological saline was inserted into the liquid container before each experiment, and the jet nebulizer was turned on and tested five times before the HELOS took a reading. The nozzle of the liquid container is located 3 cm below the center of the observation laser. All actions were launched upwards. The data are reported as the volume diameter, which is defined by 10 %, 50 % (volume median), and 90 % (X_10_, X_50_, and X_90_, respectively). The volume average particle size is defined as VMD. Triplicates measurements were taken for each sample.

### 2.4 3D printed models of sinonasal cavities

#### 2.4.1 Protocol of CT scan

A post-operative rhinosinusitis patient was imaged with CT scan. The scans were conducted on a GE LightSpeed CT scanner (GE Medical Systems, Milwaukee, WI, USA) with 0.625 mm-thick axial cuts; the intermediate window width and level settings of the CT scan were 2000 and 200 Hounsfield units, respectively. The coronal and parasagittal CT scan images were reconstructed on a computer workstation. CT data after imaging were saved in DICOM (Digital Imaging and Communications in Medicine) format. This study was authorized by the Clinical Research Ethics Committee of Peking University People's Hospital (2021PHB016-001, 2022/01/26) in accordance with the Declaration of Helsinki.

#### 2.4.2 CT data management

The DICOM images were then processed using MATLAB® software (MathWorks, Inc., Natick, Massachusetts, United States). A CT scan was combined by stacking 2D image slices to produce 3D models with 0.05-mm voxel sizes in the X, Y, and Z directions. Each slice of the image in the X-Y plane was preprocessed, and noise was removed with a square average filter. The edges of the image were emphasized with an unclear mask. The tissue types were then classified according to voxel optical density values. The area whose density value is greater than the area of aerated volume is regarded as solid, and the gray 3D model is converted into a binary model. The data was processed using Blender Foundation and saved in standard mosaic language (STL) format. Slic3r software generates tool paths for the printer before printing.

#### 2.4.3 3D printed models of sinonasal cavities

**Fig. 2:**
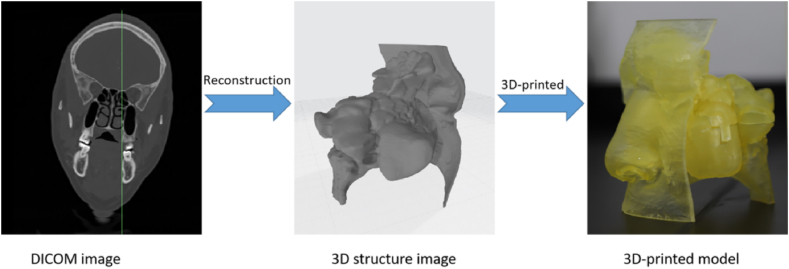
3D Printed models of sinonasal cavities.

The 3D models of sinonasal cavities (N = 24) were printed using SLASH (UNIZ Technology, United States) with a nozzle size of 0.4 mm (Fig. 2). The fused deposition modeling (FDM) printing technology offered a high resolution (10-μm layer thickness). In FDM printing, the raw material is deposited through the print head. The extruded string of molten thermoplastic material immediately joins the layer below. A commonly used thermoplastic 3D-printed acrylic (LOCTITE 3D 3840) served as the raw material. The printing is from the nasopharyngeal level above to the frontal sinus level; the printing size is 1:1. All printed models are quickly finished to make the surface of the replicas smooth. To facilitate subsequent detection, 3D-printed models of the sinonasal cavities can be broken and merged from the X–Z-axis. The 3D-printed models can be cut into several sections using a custom cutting plane. The benefit of the slicing models is to quantitatively measure the drug dose of each section, allowing for local deposition to be assessed in vitro.

### 2.5 Visualization of aerosol deposition in 3D models of sinonasal cavities

To visualize of aerosol deposition in 3D models of sinonasal cavities, the airway model is divided into two halves using a user-defined cutting plane. Sar-Gel was coated to the internal surface of the sinonasal cavities with a cotton swab to provide a uniformly coated thin layer (∼1 mm) on the 3D models of sinonasal cavities. An electronic scale was then used to measure the weight of the liquid container and liquid container with the liquid dressing. The liquid container with liquid dressing was connected to the nebulizer (BM-TCA, or PARI), and the nozzle of the liquid container was then inserted into the nostrils of the 3D models of sinonasal cavities. The nebulizer was then turned on for 30 s (n = 3), and the weight of the liquid container was immediately measured again after nebulization; 3D models of the sinonasal cavities were then constructed. Next, photos of the Sar-Gel-colored 3D models of sinonasal cavities were taken. The procedure was repeated for 60 s and 120 s of liquid dressing nebulization. The process was replicated for nebulization with saline instead of liquid dressing for 30 s, 60 s, and 120 s.

### 2.6 Quantification of aerosol deposition in regional sedimentation of the nasal cavity

#### 2.6.1 HPLC assay

**Fig. 3:**
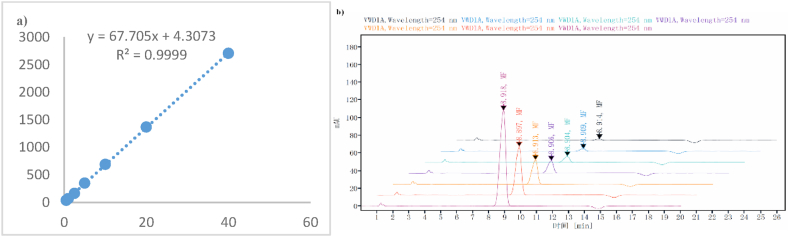
Calibration curve and chromatogram of mometasone furoate. a) calibration curve; b) chromatogram of mometasone furoate.

An Agilent 1260 Infinity II liquid chromatography system (Agilent, USA) with C_18_ column was used. The condition for gradient elution was using 1 % acetic acid aqueous solution, methanol and acetonitrile (39:13:48, v/v) with the flow rate of set at 1 mL/min. The wavelength of the UV detector was set to 254 nm, and the total operating time was 20 min. Calibration curve was developed to evaluate linearity, accuracy, and precision (Fig. 3a & b). If the measurement results were within ±15 % of the actual value (the lower limit of quantification is ±20 %), the precision and accuracy were considered acceptable. The reference substance, mometasone furoate (MF) was diluted to 0.5 μg/ml, 1 μg/ml, 2.5 μg/ml, 5 μg/ml, 10 μg/ml, 20 μg/ml, and 40 μg/ml with HPLC-grade acetonitrile for HPLC analysis. The regression equation was y = 67.705x+4.3073 with a correlation coefficient R^2^ = 0.9999 (Fig. 3a). The results indicated a linear relationship between peak area and concentration of mometasone furoate solution from 0.5 to 40 μg/mL.

#### 2.6.2 Nebulization of sample with MF

The MF powder (0.05 mg) was dissolved in 1 mL of liquid dressing. The 3D sinonasal cavities were upright and tilted slightly backwards when held by a clamp. The liquid dressing with MF was then nebulized into each nostril of the 3D sinonasal cavities for 15 s at 45° to the horizontal; the depth of nostril insertion was 5 mm. The deposition measurement of each nasal nebulization was repeated three times. Following each nebulization, the 3D sinonasal cavities were carefully disassembled into 5 parts by laser-cut with a precision of 0.3 mm. They included frontal sinus, maxillary sinus, sphenoid sinus & ethmoid sinus, turbinates, nasal vestibule & rhinopharyn as separate sections, which was capable for allows for widely tests based on the regional targeting of medication. Each model was used only once. Acetonitrile, a chromatographically pure solvent was the eluent (10 ml) used to wash each disassembled part. The resulting sample solution was used for chromatographic analysis. The analytical procedures were finished, and the concentrations of MF were calculated from the corresponding regression equations. The drug (MF) deposition referred to the percentage of drug dosages measured in each section to the total delivered dosages, which was measured by nebulization rate as discussed in the previous section.

## 3 Results

### 3.1 The particle size

**Fig. 4:**
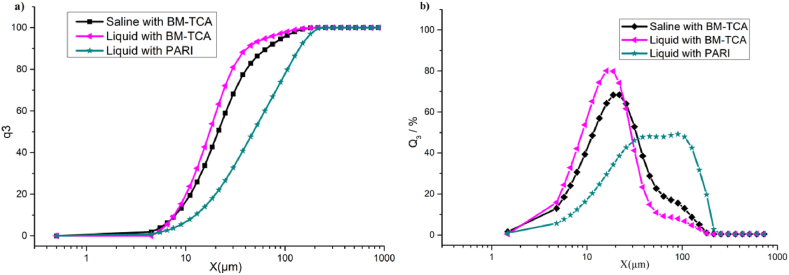
Particle size distribution of aerosolized inhalation liquid dressing. a) particle size cumulative distribution; b) particle size frequency distribution.

The particle size (N = 3) was determined by laser diffraction (Table 1). The volume percentage of particle size (>5 μm) produced by nebulization with liquid dressing through BM-TCA was 98.77 %. The volume percentage of particle size between 5 and 30 μm was 79.75 %. The volume average particle size (VMD) was 23.50 μm. However, the particle size distribution of saline after nebulization was slightly wider compared to the liquid dressing in Fig. 4a and b (Q_3_ represents particle size cumulative distribution, and q_3_ represents particle size frequency distribution). In total, 97.13 % of the particles size produced by nebulization with saline through BM-TCA were larger than 5 μm, and the volume average particle size was 30.26 μm. The volume percentage of the particle size between 5 and 30 μm was 65.23 %, which is less than the liquid dressing at the same size range. The volume percentage of particle size (>5 μm) produced by the nebulizer (PARI) was 33.56 %. The volume percentage of particle size between 5 and 30 μm was 33.56 %.

**Table 1:** Particle size of liquid dressing and saline produced by nebulization.

Samples	x10	x_50_	x90	VMD	>5 μm	5–30 μm
	(μm)	(μm)	(μm)	(μm)	(%)	(%)
Liquid dressing through BM-TCA	7.67 ± 0.03	17.46 ± 0.05	41.58 ± 0.07	23.50 ± 0.07	98.77 ± 0.03	79.75 ± 0.02
Saline through BM-TCA	7.87 ± 0.04	21.21 ± 0.01	65.20 ± 0.02	30.26 ± 0.09	97.13 ± 0.09	65.23 ± 0.08
Liquid dressing through PARI	1.14 ± 0.02	3.71 ± 0.04	9.22 ± 0.05	4.51 ± 0.08	33.56 ± 0.01	33.56 ± 0.05

X_10_: The particle size where 10 % of the total volume below this value).

X_50_: The particle size where 50 % of the total volume below this value.

X_90_: The particle size where 90 % of the total volume below this value).

VMD: volume median diameter.

### 3.2 Nebulization rate

The average nebulization rate for liquid dressing (through BM-TCA) was 0.70 ± 0.05 g/min, and for saline (through BM-TCA) was 0.72 ± 0.09 g/min (mean ± SD, n = 3) (Table 2). There was no obvious difference between the three measurements. The calculated nebulization rate for liquid dressing was 0.67 ± 0.05 ml/min based on the densities of the liquid materials. The nebulization rate for saline (through BM-TCA) was 0.72 ± 0.09 ml/min. The nebulization rate for liquid dressing (through PARI) was 0.67 ± 0.09 ml/min. The nebulization rate of BM-TCA was slightly larger than PARI.

**Table 2:** Nebulization rate of liquid dressing and saline.

Samples	Nebulization rate (g/min)	Density	Nebulization rate (ml/min)
Liquid dressing through BM-TCA	0.70 ± 0.05	1.05 g/ml	0.67 ± 0.05
Saline through BM-TCA	0.72 ± 0.09	1.0 g/ml	0.72 ± 0.09
Liquid dressing through PARI	0.65 ± 0.04	1.05 g/ml	0.62 ± 0.04

### 3.3 Visualization of Sediment distribution

**Fig. 5:**
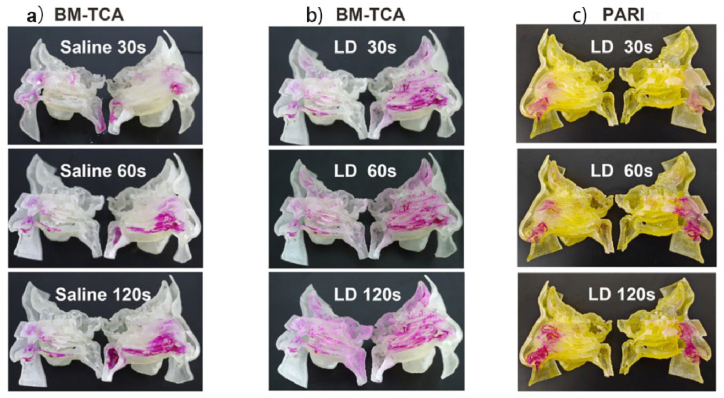
Visualized deposition. Distribution of the nasal cavity with 30s, 60s and 120s nebulization time. a) The normal saline nebulization through BM-TCA; b) The liquid dressing (LD) nebulization through BM-TCA; c) the liquid dressing nebulization through PARI. The color difference of the model in the figure is caused by the different content of aerosol, which is used to distinguish the two groups of tests.

Sar-Gel is a highly sensitive water indicating paste. When contacting to the water, the color of Sar-Gel changes from white to purple. The Sar-Gel color-changing glue helped to visualize the deposition and distribution of the liquid dressing in the nasal cavity. The deposition and distribution of the liquid dressing in the nasal cavity could be determined by observing the color-changing area (Fig. 5). The liquid dressing (through BM-TCA) was mainly deposited in the ethmoid sinus and the frontal sinus (Fig. 5b). The darkest part was the ethmoid sinus. However, there was no color change in the rhinopharynx, thus indicating that the liquid dressing was unable to enter the throat through the rhinopharynx after nebulization. There were no obvious color changes in ethmoid sinus and frontal sinus after the normal saline nebulization (through BM-TCA) in Fig. 5a. The normal saline was mainly deposited in the nasal vestibule and rhinopharynx. Similarly, the liquid dressing (through PARI) was deposited mainly in the nasal vestibule and rhinopharynx (Fig. 5c). There were no apparent color changes in the ethmoid or frontal sinuses.

### 3.4 Fraction of regional sinonasal deposition

Mometasone Furoate (MF) is a new fluoride-free glucocorticoid derivative with high efficacy and few adverse reactions. It plays a crucial role in alleviating symptoms of sinusitis, nasal polyps, seasonal and perennial allergic rhinitis. According to the standard curve, the concentration of mometasone furoate in liquid dressing suspension was measured to be 0.48 mg/ml. The liquid dressing containing mometasone furoate was nebulized for 1 min with 321.6 μg (0.48 mg/ml∗0.67 ml/min ∗1min) of mometasone furoate being emitted, according to the nebulization rate of liquid dressing. Table 3 shows the MF deposition fractions in different parts of the 3D nasal cavity after liquid dressing with mometasone furoate through the compression nebulizer (BM-TCA or PARI). The resulted percentages are the average and standard deviation of the experimental data with the 3D nasal cavities. The percent in different parts of the 3D nasal cavity (liquid dressing through BM-TCA) was equal to 41.80 ± 0.95 %, 0.70 ± 0.37 %, 14.00 ± 0.69 %, 34.70 ± 0.96 %, and 0.60 ± 0.62 % for frontal sinus, upper maxillary sinus, sphenoid sinus/ethmoid sinus, turbinates and nasal vestibule/rhinopharynx, respectively (mean ± SD, n = 3). The percent in different parts of the 3D nasal cavity (liquid dressing through PARI) was equal to 1.31 ± 0.71 %, 2.11 ± 0.81 %, 3.81 ± 0.93 % and 73.46 ± 2.02 % for frontal sinus, sphenoid sinus/ethmoid sinus, turbinates and nasal vestibule/rhinopharynx, respectively. The deposition fractions of upper maxillary sinus of the 3D nasal cavity after liquid dressing through PARI were not detected. These results are close to the results of the previous visualized deposition distribution of the liquid dressing. So the aerosol deposition is an effective method to deliver steroids to the turbinates and frontal sinus. Therefore, it is feasible to use HPLC to quantitatively analyze the deposition and distribution of mometasone furoate-containing liquid dressings in the nasal cavity.

**Table 3:** The deposition fraction of mometasone furoate liquid dressing in different parts of the nasal cavity defined as the percentage distribution of metered dose (MF %).

Distribution area	Percentage of deposition (MF %)^a^	
	Liquid dressing through BM-TCA	Liquid dressing through PARI
Frontal sinus	41.80 ± 0.95	1.31 ± 0.71
Maxillary sinus	0.70 ± 0.37	/
Sphenoid sinus, ethmoid sinus	14.00 ± 0.69	2.11 ± 0.81
Turbinates	31.70 ± 0.96	3.82 ± 0.93
Nasal vestibule, Rhinopharynx	0.60 ± 0.62	73.46 ± 2.02
Loss	11.20 ± 0.32	20.20 ± 1.67

C_local_ (μg/ml): The concentration of mometasone furoate in local areas measured by HPLC.

10 ml: The eluent volume of acetonitrile used to wash the local areas.

DF_total_: The total weight of mometasone furoate being emitted by nebulizer in 1 min.

C_MF_ = 0.48 mg/ml: The concentration of mometasone furoate in liquid dressing suspension.

NR(ml/min): Nebulization rate of liquid dressing through BM-TCA or PARI.

a MF% = C_local_ ∗ 10 ml/DF_total_ ∗ 100 %; DF_total_ = C_MF_ ∗ NR ∗ 1min.

## 4 Discussion

Many reports have been devoted to the study of drug deposition in various ways. In vitro deposition measurement results of 3D printed nasal casts can better demonstrate the nebulization characteristics of the nasal cavity. Nasal models obtained by combining CT and 3D-printed technology enable the fabrication of complicated and biomimetic models [26,30]. Then, qualitative or quantitative data can be generated depending on the model used. A good visualization of a liquid preparation deposition can be obtained by coating the airway replica with a water-indicating paste (Sar-Gel®) [27,28]. Imaging equipment and photoshop software were used to acquire quantification data of deposit areas in a single mold. Spectroscopy [[31], [32], [33]], HPLC [[34], [35], [36]] and also other techniques [[37], [38], [39]] have been used to determine the spray deposition in the olfactory region through the nasal cavities. However, there is currently no qualitative or quantitative approach for assessing in vitro distribution which suggests accurate regional distribution of drug in sinus cavities without the use of radioactive isotopes or high-energy radiation [40,41].

The lesions of sinusitis are generally located in the four anatomical sinus cavities of the sinuses, the maxillary sinus, ethmoid sinus, frontal sinus and sphenoid sinus on both sides. Oral antibiotics, nasal irrigations, systemic and topical corticosteroids are currently recommended medical treatment options. Local administration has become an indispensable method for the treatment of CRS. Clinical studies have pointed out that the frontal sinuses and turbinates area are important sites for clinical medication [13,42]. For patients with ineffective drug treatment, functional endoscopic sinus surgery (FESS) is advised. Local treatment of steroids is also the mainstream method of permanently effective treatment after operation [43,44]. Researches have indicated that the extensive operation and the size of the ostia enlargement are positively correlated with the ratio of drug deposited into the sinus cavity [43]. Due to the complex anatomy of the sinuses, local drug administration to the sinuses remains very difficult. The frontal and maxillary sinuses are connected almost vertical to the majority of the airflow in the nasal cavity, which pose additional difficulties to local deposition [[45], [46], [47]]^.^

According to the marketed drug delivery systems, drops, sprays, irrigations, and more recently nebulizers are the main types of nasal preparations. Compared with nasal spray, local aerosol inhalation of glucocorticoid suspension has a larger range of action and longer drug residence time, which is an effective way to inhibit the growth of nasal polyps and relieve patients' symptoms [48]. In the regional deposition systems, nasal nebulizers have gradually become the most potential pharmaceutical delivery technique for regional and systemic treatments. However, for the frontal sinus region, nasal aerosol inhalation still has deficiencies of relatively low drug concentration in the frontal sinus region, and the operation is relatively complicated. It is known that there are multiple key factors that can affect the delivery of nebulized materials. Particle size has continuously been considered as the most significant factor affecting particle deposition in the nasal cavity [36,49,50]. The particle size of 1–5 μm benefits the lower respiratory tract [51]. Particles with a diameter over >5 μm are mainly deposited in the nasal cavity and sinonasal cavity [52]. Traditional jet and vibrating mesh nebulizers (such as PARI) are designed specifically for delivering medications to the lungs; therefore, these devices generate respirable particles in the 3-5micron size range in order to reach the lung mucosa, regardless of the user's choice of oral or nasal inhalation. For sinonasal deposition, the optimal particle size should be significantly larger than the respirable particle size, i.e., larger than 5 μm [53]. Obviously, the particle size of the liquid dressing through the BM-TCA nebulizer is greater than that through PARI nebulizer. Nearly 79.75 % of particle sizes are between 5 and 30 μm. The unique delivery characteristics of the BM-TCA nebulizer results in broad intranasal drug delivery with high intranasal drug retention. PARI is a small particle nebulizer, and the particle size was still small despite using the same liquid dressing. 33.56 % of particle sizes are between 5 and 30 μm. Most particle sizes are (63.44 %) <5 μm. The volume average particle size (VMD) is 4.5 μm; this is much smaller than the particle size of the liquid dressing based on BM-TCA (VMD = 23.50 μm).

When the liquid dressing is combined with the air flow generated by small particle nebulizer (PARI), the particles will remain in the nasal vestibule, rhinopharynx. When the saline was combined with the air flow generated by BM-TCA, they will remain mostly in the nasal vestibule, ethmoid sinus, rhinopharynx. The large particles of the liquid dressing will be distributed throughout the nasal cavity including the clinically important parts (frontal sinus and turbinates) when combined with the air flow generated by the medical compressed nebulizer (BM-TCA; Fig. 5). The area reached by the liquid dressing includes the frontal sinuses, sphenoid recesses, ethmoid sinuses, sphenoid/maxillary sinuses, all turbinates, middle nasal passages, and olfactory. The liquid dressing aerosol is captured by the nasal cavity and sinus cavity and deposited in the entire nasal cavity and sinus cavity. The nebulizer delivers the liquid dressing to the target site while reducing the deposits in the rhinopharynx, trachea, bronchus, and other parts, thus avoiding the risk of lung deposits and related side effects. Consequently, the nebulizer and formulation factors were regarded as the key parameters affecting particle size distribution.

Nasal sprays and small particle nebulizers targeted mainly to the anterior areas, with low distribution of drug into the posterior nasal passages, which included regional sections, such as the four anatomical sinuses and turbinates. Shah's group [54] firstly reported the quantification of local deposition by using a suspension preparation, with 60 % deposition in the posterior areas (in 15min). The experiments didn't analyze smaller areas and quantitatively evaluate the local distribution of drugs in each sinus region. Siu's group [55] suggested that pressurised metered dose inhalers (MDI) produced better results than nasal spray, with a larger percentage of total delivery to the sinuses (0.7 in the frontal sinuses, 11.1 % in the sphenoid and ethmoid sinuses, 1.7 % in the maxillary sinuses). In healthy subjects, the total deposition in the maxillary sinus and sphenoid sinus (estimated by planar gamma camera imaging) after pulsed aerosol infusion was 9.7 %. The percentage of deposition in the frontal sinus was less than 1. Due to the extremely narrow drainage channel of the frontal sinus, these preparations were still difficult to reach the frontal sinus. The reason for the poor sinus deposition and efficacy of nasal sprays was the delivery of large particles with high velocities [41,56], which were deposited in the anterior nasal cavity [[57], [58], [59], [60]], thus unable to reach the affected sinus mucosa through the narrow passages.

The effectiveness of this study depends on the verification of nasal replica, which confirms that the initial visualized deposition indicates the delivered locations of liquid dressing suspension with MF. Similar to the results reported in an earlier study using PARI nebulizer [61], all the emission dose will not be deposited in the nasal cavity through the pulsed aerosol technology. The main deposits are the nasal vestibule and the rhinopharynx (73.46 %). The experimental results of aerosol deposition in the nasal cavity prove that the generated aerosol has excellent performance with larger particle size (23.5 μm VMD of the BM-TCA compressor versus 4.5 μm VMD of the PARI nebulizer, vibrent prototype). The aerosol with larger particles can be deposited in the sinus areas located at the posterior regions of the nasal cavity. The BM-TCA nebulizer generally results better outcomes under the same circumstances, accounting for a larger percentage of regional deposition within the turbinates (34.70 %) and the sinuses (up to 41.80 % in the frontal sinuses, up to 14.00 % in the sphenoid and ethmoid sinuses, 0.70 % in the maxillary sinuses). To date, this is the maximum deposition of aerosols within the frontal sinuses. Improvements in local nasal deposition are obtained after patients undergo more thorough FESS [25,62].

## 5 Limitations of this study

Although the results obtained in this study are valuable, the use of rigid nasal models may affect the authenticity of in vitro results. These models of nasal cavity did not take into account individual differences, including gender, age, height, weight, and race. The constant flow was used instead of the actual breathing modes. The rapid mucociliary clearance was not considered in this study. Because of the obvious differences between nasal passages geometry and inhalation patterns in patients with sinusitis and normal adults, the dosages expected to achieve the target will be different. Obviously, larger sample groups are needed to minimize the individual differences in each age group.

## 6 Conclusion

In conclusion, an effective nasal nebulization evaluation approach has been proposed for visualizing and quantifying regional deposition. This has been used to compare the deposition patterns of two different commercially available nasal drug delivery devices: BM-TCA and PARI. Deposition experiments using 3D-printed nasal casts are suitable for in vitro performance tests, comparing various nasal devices, preparations, and deposition protocols in diverse experimental environments. Moreover, they are very useful for accurately characterizing nasal drug deposition by camera and HPLC in specific areas of the nose. Since specific addition of dyes or radioactive labels were not required, this technique has obvious advantages over flat-panel or laser-based spray mode imaging and scintillation imaging. Although the nasal spray is currently the more widely used method for CRS, particularly in the postoperative setting, the nasal nebulizer is a newly developing technology which seems to cover the sinuses of the nasal cavities well, indicating that it would be an effective technique for the local drug delivery. Manufacturers can improve local treatment through equipment and preparation techniques to achieve the desired treatment of nasal nebulizers. Since these studies were conducted in 3D models, further study in diseased patients is worth merited to better understand nasal deposition and its relationship to efficacy and safety. Prospective researches in patients with CRS will be helpful to further clarify the clinical equivalence of in vitro studies.

## Institutional review board statement

This study was authorized by the Clinical Research Ethics Committee of Peking University People's Hospital (2021PHB016-001, 2022/01/26) in accordance with the Declaration of Helsinki.

## Data availability statement

All data generated or analyzed during this study are included in this published article.

## Funding

The authors disclosed receipt of the following financial support for the research. This study was supported by The 10.13039/100007831Capital Health Development Foundation (No: 2020-1-2051) and 10.13039/501100007937Peking University People's Hospital Scientific Research Development Funds (RDL2021-05).

## CRediT authorship contribution statement

**Wei He:** Writing – original draft, Visualization, Validation, Methodology, Investigation, Formal analysis, Data curation. **Muhan Shi:** Resources, Formal analysis, Data curation. **Yaozhong Lu:** Software, Data curation. **Chengsheng Chu:** Validation, Formal analysis. **Xiaolong Wang:** Visualization, Software. **Min Wang:** Resources, Funding acquisition. **Xiaofang Zhang:** Writing – review & editing, Supervision, Project administration, Conceptualization.

## Declaration of competing interest

The authors declare that they have no known competing financial interests or personal relationships that could have appeared to influence the work reported in this paper.
